# Long non-coding RNA SOX2OT: expression signature, splicing patterns, and emerging roles in pluripotency and tumorigenesis

**DOI:** 10.3389/fgene.2015.00196

**Published:** 2015-06-17

**Authors:** Alireza Shahryari, Marie Saghaeian Jazi, Nader M. Samaei, Seyed J. Mowla

**Affiliations:** ^1^Stem Cell Research Center, Golestan University of Medical Sciences, Gorgan, Iran; ^2^Department of Molecular Medicine, Faculty of Advanced Medical Technologies, Golestan University of Medical Sciences, Gorgan, Iran; ^3^Department of Medical Genetics, Faculty of Advanced Medical Technologies, Golestan University of Medical Sciences, Gorgan, Iran; ^4^Department of Molecular Genetics, Faculty of Biological Sciences, Tarbiat Modares University, Tehran, Iran

**Keywords:** lncRNA, SOX2OT, splicing pattern, expression signature, pluripotency, tumorigenesis, stem cell

## Abstract

SOX2 overlapping transcript (SOX2OT) is a long non-coding RNA which harbors one of the major regulators of pluripotency, SOX2 gene, in its intronic region. *SOX2OT* gene is mapped to human chromosome 3q26.3 (Chr3q26.3) locus and is extended in a high conserved region of over 700 kb. Little is known about the exact role of SOX2OT; however, recent studies have demonstrated a positive role for it in transcription regulation of *SOX2* gene. Similar to SOX2, SOX2OT is highly expressed in embryonic stem cells and down-regulated upon the induction of differentiation. SOX2OT is dynamically regulated during the embryogenesis of vertebrates, and delimited to the brain in adult mice and human. Recently, the disregulation of SOX2OT expression and its concomitant expression with SOX2 have become highlighted in some somatic cancers including esophageal squamous cell carcinoma, lung squamous cell carcinoma, and breast cancer. Interestingly, SOX2OT is differentially spliced into multiple mRNA-like transcripts in stem and cancer cells. In this review, we are describing the structural and functional features of *SOX2OT*, with an emphasis on its expression signature, its splicing patterns and its critical function in the regulation of *SOX2* expression during development and tumorigenesis.

## Introduction

According to the recent genome-wide studies, most of the human genome is transcribed, yielding a complex network of large and small RNA molecules in human cells. However, only 1–2% of the transcripts have the capacity for protein translation (Kapranov et al., 2007; Guttman et al., 2009). The new class of long (or large) non-coding RNAs (lncRNAs) comprises the most proportion of the human transcriptome. Little is known about the exact functional roles of lncRNAs in human. Nevertheless, some recent studies have reported dysregulations of lncRNAs in several human disorders. LncRNAs key roles in the regulation of pluripotency, stem cells differentiation, and tumorigenesis are emerging (Perez et al., 2007; Gupta et al., 2010; Loewer et al., 2010; Esteller, 2011; Ng et al., 2011; Prensner and Chinnaiyan, 2011). Furthermore, a number of studies have achieved toward a therapeutic effect for some genetic disorders by targeting an lncRNA *in vitro* and *in vivo* (Gupta et al., 2010; Gutschner et al., 2013; Meng et al., 2015).

SOX2 is a HMG-box transcription factor which is essential for the maintenance of self-renewal and the pluripotency of undifferentiated embryonic stem cells (Avilion et al., 2003; Fong et al., 2008). More interestingly, SOX2 along with OCT4, c-Myc and Klf4 plays a critical role in the generation of induced pluripotent stem cells (iPSC) from both adult human and mouse somatic cells (Takahashi and Yamanaka, 2006; Takahashi et al., 2007). Recently, it has been suggested that SOX2 promotes tumor initiation and controls cancer stem cell properties in squamous cell carcinoma (SCC) of the skin tumors (Boumahdi et al., 2014). The single-exon *SOX2* gene was mapped to the human chromosome 3q26.3 (Chr3q26.3) locus, where it is embedded within the intronic region of a multi-exon lncRNA, known as *SOX2 overlapping transcript (SOX2OT)* which transcribed in the same orientation as *SOX2* (Fantes et al., 2003). While little is known about the exact role of *SOX2OT*, recent studies have demonstrated a positive role for it in the regulation of *SOX2* gene in human stem cells (Amaral et al., 2009; Shahryari et al., 2014).

Human *SOX2OT* gene has a high nucleotide identity with its ortholog in mouse and other vertebrates, demonstrating its high degree of evolutionarily conservation. The multi-exon SOX2OT has no open reading frame (ORF), but is spliced into several mRNA-like transcripts with the longest one of approximately 3.5 kb in human (Amaral et al., 2009; Shahryari et al., 2014). Close concomitant expression patterns of SOX2OT and SOX2 in stem cells and some human cancers, have all suggested that they may be co-regulated and involved in similar molecular pathways. Accordingly, some recent reports have demonstrated the transcriptional regulation of *SOX2* by SOX2OT (Amaral et al., 2009; Askarian-Amiri et al., 2014; Hou et al., 2014; Shahryari et al., 2014).

In this review, we have delineated the complex structure and functional features of *SOX2OT* locus, with more emphasis on its expression and splicing patterns, and its potential role in the regulation of SOX2 expression during the development and cancer progression.

## Genomic Architecture of *SOX2OT* Gene Region in Vertebrates

**FIGURE 1:**
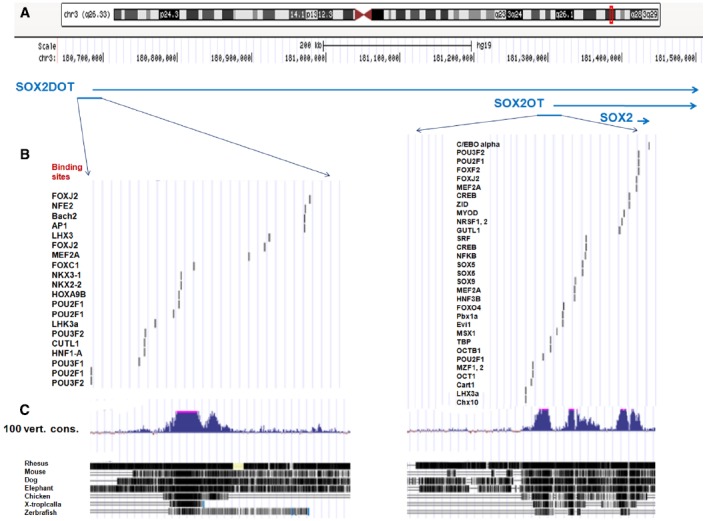
**Genomic architecture of Chr3q26.33 region in human and vertebrates. (A)** The banding pattern of chromosome 3 and location of *SOX2OT* locus of 3q26.33 is presented according to the UCSC genome browser (h19 assembly). **(B)** The conserved transcription factor binding sites is presented at upstream of human genomic regions of SOX2OT and the isoform of SOX2DOT. The binding sites distribution for multiple transcription factors of POU domain and HMG-box families is noticeable. **(C)** A high degree of conservation at upstream of genomic regions of SOX2OT and SOX2DOT in 100 vertebrates is presented, using Multiz alignment program (adopted from http://genome.ucsc.edu).

*SOX2OT* gene with the official symbol of *SOX2-OT* (also known as NCRNA00043) was originally mapped to the human chromosome 3q26.3-q27 locus [current location of NC_000003.12 (181056680..181742228)], and harbors one of the main regulators of pluripotency, *SOX2* gene [also known as *ANOP3* and *MCOPS3*, with current location of NC_000003.12 (181711924..181714436)], in its intronic region overlapping in the same transcriptional orientation (Fantes et al., 2003). *SOX2OT* gene is located and extended in a highly conserved region of over 700 kb in human and other vertebrates (Fantes et al., 2003; Amaral et al., 2009; Figure 1A).

Amplification of several genomic regions at 3q26-qter chromosome is associated with multiple human cancers (Massion et al., 2002; Jiang et al., 2004). The gene amplification events in those regions, particularly in q26–q29 region of chromosome 3, are present in the multiple types of SCCs of different tissues including lung, head and neck, esophagus, and cervix (Gebhart and Liehr, 2000; Balsara and Testa, 2002; Bass et al., 2009). Interestingly, a 2 Mb gained/amplified genomic region in 3q26.3 which encompasses *SOX2* and *SOX2OT* has been reported in lung SCC (Hussenet et al., 2010).

Chromatin modification maps of chromosome 3q26.3-q27 acquired by chromatin immune precipitation sequencing (ChIP-Seq) data represented several transcription start sites (TSSs) for *SOX2OT* gene (Mikkelsen et al., 2007; Amaral et al., 2009). Those promoter regions embedded within 1–7 non-coding highly conserved sequence blocks in vertebrates known as highly conserved elements (HCEs), probably are associated with the regulatory region of *SOX2OT*. These blocks of transposon-free regions with over 5 kb long have remained resistant to transposon invasion throughout vertebrate evolution and encompassed regulatory sequences controlling the expression of genes that are involved in early development (Simons et al., 2006; Amaral et al., 2009).

Interestingly, analysis of the alternative TSSs of *Sox2ot* orthologous in various vertebrates demonstrated the existence of a distal promoter, located over 500 kb upstream of the *SOX2OT* sequence in mouse and human (*SOX2OT* refers to human and *Sox2ot* to non-human). This distal promoter region which is associated with the transposon-free region, highly positional conserved elements, and histone modification marks of promoters, created a novel isoform of Sox2ot termed Sox2dot (Sox2 distal overlapping transcript) which has HCE 1 with an enhancer-like function in the mouse’s developing forebrain (Amaral et al., 2009). Here, we bioinformatically analyzed the potential binding sites for transcription factors in a highly conserved genomic regions upstream of *SOX2OT* and *SOX2DOT*. As illustrated in Figure 1, the data represent the existence of binding sites of several transcription factors involved in cancer progression as well as stem cells pluripotency and differentiation in those regulatory regions. Noticeably, the number and distribution of binding sites of some transaction factors belonging to POU domain and HMG-box families is surprising (Figures 1B,C).

**FIGURE 2:**
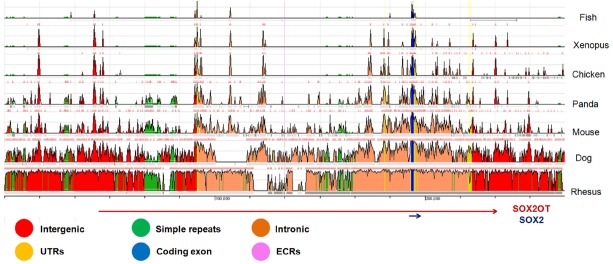
**A schematic representation of comparative genomics locus of *SOX2OT* in different species.** The evolutionary conserved regions (ECR) of chr3:181236624-18150288 adapted from ECR browser (http://ecrbrowser.dcode.org) on human (hg19) is presented. The data is compared with some well-known vertebrates including rhesus, dog, mouse, chicken, *Xenopus*, and fish. The ECR length and similarity considered as browser default. The UCSC known genes of *SOX2OT* and *SOX2* locating in the region is shown at the bottom. Notice that the most conserved regions are concentrated around SOX2 overlapping region.

Primary sequence analysis of *sox2ot* in vertebrates including fish, reptiles, amphibians and mammals highlighted some highly conserved regions, including a 400-nt segment in exons near to *SOX2* gene, as well as an upstream region with more than 90% identity between mouse and human genomes. However, there is only a low degree of conservation when full length sequence of *SOX2OT* gene (∼750 kb) is compared among different species (Amaral et al., 2009; Figure 2).

## Splicing Patterns of *SOX2OT* Gene in Human and Other Vertebrates

Protein-coding capacity parameters including ORF length, synonymous versus non-synonymous base substitution rates, and similarity to known proteins demonstrated that human and mouse SOX2OT/Sox2ot full-length sequences have no significant protein-coding potential. Nevertheless, there is a possibility for generation of some small peptides, encoded by some transcripts (Dinger et al., 2008; Amaral et al., 2009). Mark signs of mRNAs including a lot of cap and poly Adenine signals suggest that *SOX2OT* gene is transcribed by RNA polymerase II enzyme, and produces a mRNA-like lncRNA transcript (Numata et al., 2003; Amaral et al., 2009).

Human and mouse SOX2OT have multiple TSSs, and several alternatively spliced variants and polyadenylation sites have already been reported for them (Amaral et al., 2009). Several full-length clones of mouse sox2ot have been registered with a wide range of sizes, from 638 nucleotides (GenBank accession no. BY721402) to an approximately 3.5 kb form (accession no. AK031919). The various sizes of the registered cDNA clones are in accordance with the Northern blot data obtained from several mouse tissues. While the most abundant isoform of sox2ot posses a size of ∼3 kb, several other rare ones with approximate sizes of 1, 4, 6, and >10 kb have also been reported in some mouse tissues. In zebrafish embryo, Northern blot analysis revealed an abundant 2.5 kb transcript variant and two other less abundant transcripts of 1.5 and 6 kb (Amaral et al., 2009).

As we have previously reported, SOX2OT is spliced into several transcript variants, including SOX2OT, SOX2OT-S1, and SOX2OT-S2 which co-upregulated with master regulators of pluripotency, SOX2 and OCT4, in esophageal squamous cell carcinoma (ESCC). SOX2OT-S1 (Accession no: JN711430, GI: 379031002) lacks exon 4 of the main transcript, whereas SOX2OT-S2 (SOX2OT-S2; Accession no: JN882275, GI: 379031003) lacks exons 3 and 4. In addition to the experimentally approved novel transcripts, human EST database (dbEST) also provided some ESTs with GenBank accession numbers BX423294.2, BX442540.2, BX459910.2, DA268964.1, and DA282731.1 which are related to the novel sequence of exon 3-exon five junction in SOX2OT-S1, and DA308672.1 which is related to the novel sequence of exon 2-exon five junction in SOX2OT-S2 variant (Shahryari et al., 2014).

**FIGURE 3:**
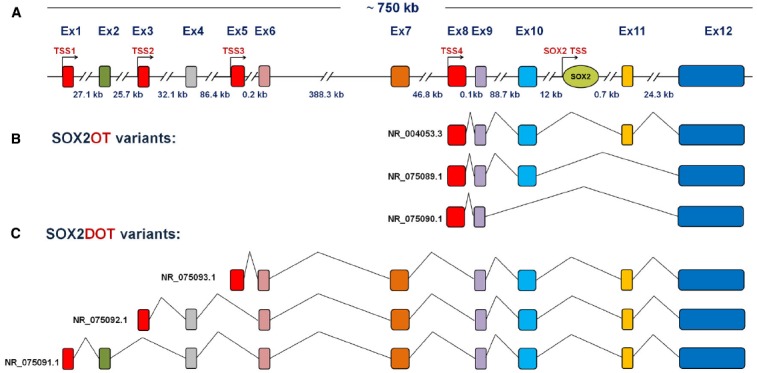
**Schematic representative of human *SOX2OT* gene and its splicing patterns. (A)** Multi-exon gene of SOX2OT is located on human Chr3q26.3 and is extending in a 750 kb genomic region, where it holds the single-exon of SOX2 gene in its intronic region in the same strand and orientation. **(B)** The splicing patterns of SOX2OT and **(C)** SOX2DOT isoforms are presented, respectively.

More than 15 different *Major Class* of introns (GT-AG), at least 13 spliced variants, and six TSSs were presented for SOX2OT using bioinformatics analysis and AceView annotation (Amaral et al., 2009). Our group has been also identified several novel variants of SOX2DOT, which demonstrates a complex pattern of TSSs and alternative splicing of SOX2OT (Figure 3A). According to the validated NCBI Reference Sequence (RefSeqs), splicing patterns of SOX2OT, as illustrated in Figure 3, generates at least six transcript variants. Among those, three variants are generated from alternative splicing of SOX2OT, while the other three ones are originated from SOX2DOT (Figures 3B,C).

## Expression Signature of SOX2OT in Somatic, Stem, and Cancer Cells

Sox2ot isoforms are widely expressed in whole embryo and newborn mouse, but in adult tissues their expression is primarily restricted to brain. It is also expressed at lower levels in tissues where Sox2 is also expressed, such as lung, as well as in tissues were sox2 is not expressed, such as testis. Nevertheless, sox2dot isoform is exclusively expressed in adult mouse brain tissues. Concomitant with Sox2, Sox2ot is mainly expressed in mouse embryonic stem cells and down-regulated during the course of differentiation. Nevertheless, only Sox2ot is upregulated during the late mouse embryoid body differentiation events. Moreover, expression of Sox2 and Sox2ot are coregulated during mouse neurosphere differentiation *in vitro*. Accordingly, Sox2dot isoform is also upregulated upon the induction of differentiation in neurospheres. Similar to mouse, Sox2ot and sox2 are also dynamically regulated during embryogenesis of other vertebrate, including chicken and zebrafish (Mercer et al., 2008; Amaral et al., 2009).

The lncRNA SOX2OT is co-upregulated with master regulators of pluripotency, SOX2 and OCT4, in ESCC. The qRT-PCR analysis revealed a high level of SOX2OT expression in tumor samples of ESCC, compared to the apparently non-tumor marginal tissues from the same patients, which suggested a potential part for it in tumorigenesis of esophagus (Shahryari et al., 2014).

A concomitant expression pattern of *SOX2OT* with that of *SOX2* and *OCT4* genes is reported in a pluripotent cell line, NT2. SOX2OT and its variants also proved to have a distinct expression pattern during neural differentiation of NT2 cells. The expression pattern of SOX2OT variants was similar to those of SOX2 and OCT4, and downregulated upon the induction of neural differentiation. However, in contrast to a complete shut-down of SOX2 and OCT4 expression, a low expression of SOX2OT and its variants is persisted in later time points of differentiation (Shahryari et al., 2014).

Distinct differences in the expression patterns of SOX2OT and SOX2 were observed in breast cancer tissue samples. Analysis of the genome-wide RNA transcript profiles from the Cancer Genome Atlas (breast invasive carcinoma gene expression) by RNA Seq data set in 1106 samples of breast cancer tissues revealed the concordant expression of SOX2OT and SOX2 in this somatic cancer. SOX2OT and SOX2 are highly expressed in estrogen receptor positive (ER+) breast cancer cell lines, in comparison with the ER– ones. In ER+ breast cancer cell lines, expression of SOX2OT is positively correlated with SOX2 expression level, albeit at lower levels. Moreover, SOX2OT and SOX2 are co-upregulated in suspension culture conditions of breast cancer cell lines which advocates the growth of cellular subpopulation with cancer stem cell-like properties (Askarian-Amiri et al., 2014).

Overexpression of both SOX2OT and SOX2 has been reported in human primary lung cancer tissues, in comparison with the corresponding non-tumor samples. Furthermore, SOX2OT demonstrated a significant high expression level in SCC of the lung, compared with adenocarcinoma ones. There was a positive correlation between SOX2OT and SOX2 expression levels in the same lung cancer tissue samples (Hou et al., 2014).

**FIGURE 4:**
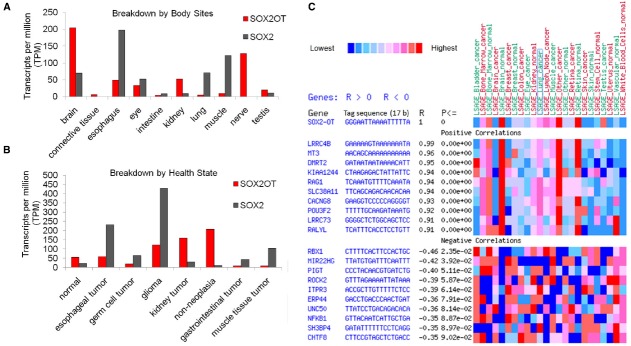
**Expression signature of SOX2OT and its correlations with other genes in human. (A,B)** Approximate expression profiles of SOX2OT and SOX2 based on ESTs (dbEST) distribution in human tissues, and tumors libraries; the data is presented according to TPM (transcript per million) in pool. High enrichment of SOX2OT expression in CNS libraries and also brain-derived tumors is noticeable. **(C)** Two dimensional display of gene expression summary of SOX2OT SAGE expression represents positive and negative significant correlation with multiple key genes involved cancer progression, stem cell pluripotency, and development. The data was adopted from available SAGE libraries of the cancer genome anatomy project database [Cancer Genome Anatomy Project (CGAP)] (http://cgap.nci.nih.gov). Ten positively correlated genes (*R* > 0.9 top of map) and 10 negatively correlated ones (*R* < –0.35, bottom of map) are listed (*P*: *P* value, *R*: correlation value).

In order to expand our knowledge of expression regulations, we reviewed some resources on gene expression profile of SOX2OT and SOX2. Exploring the expressed sequence tags (ESTs) profiles which are available from NCBI, demonstrated the expression patterns of SOX2 and its overlapping transcript in multiple pools of different human tissues and tumors. The data represent the possibility of SOX2OT and SOX2 expression in a wide list of human tissues including brain, connective tissue, esophagus, eye, intestine, kidney, lung, muscle, nerve, and testis. More interestingly, the data hint the possibility of upregulation of SOX2OT expression in glioma and kidney tumors. In agreement with the data reported by Amaral et al. (2009) our results also revealed a high enrichment of SOX2OT expression in CNS libraries (Figures 4A,B). The high expression of SOX2OT and some other lncRNAs in CNS tissues suggests a potential role for them in animal brain development and function (Amaral and Mattick, 2008).

We also evaluated cancer genome anatomy project resources [Cancer Genome Anatomy Project (CGAP)] to find out a correlation between the expression signatures of *SOX2OT* with that of other genes. Based on SAGE (Serial Analysis of Gene Expression) data, SOX2OT represented a significant positive and negative correlation with multiple key genes involved in neuronal development (e.g., *LRRC4B*) addressing its function in CNS development. Furthermore, cancer associated genes (e.g., *ROCK2*, *NFKB*) are also significantly correlated with SOX2OT in SAGE libraries; which highlighted the potential function of SOX2OT in cancer progression. Noticeably, a significant positive correlation of *POU3F2* transcription factor which had multiple binding sites in genomic regulatory region of SOX2OT was observed (Figure 4C).

## The Potential Roles of SOX2OT in Pluripotency and/or Tumorigenesis Through Regulation of SOX2 Expression

Transcription factor SOX2 regulates the expression of more than one thousand genes in stem cells where small changes of its expression strikingly alter the self-renewal and pluripotency properties; hence SOX2 acts role as a molecular *rheostat* in those cells (Boyer et al., 2005; Boer et al., 2007; Kopp et al., 2008; Amaral et al., 2009; Mandalos et al., 2014). Recent evidences have demonstrated that gene amplification and/or aberrant expression level of SOX2 play a role in the development and tumorigenesis of many types of cancer including pancreatic carcinoma, prostate, breast, lung, gastric, and esophagus cancers (Gure et al., 2000; Sattler et al., 2000; Sanada et al., 2006; Rodriguez-Pinilla et al., 2007; Chen et al., 2008; Jia et al., 2011; Hütz et al., 2013). SOX2 is also involved in the proliferation and anchorage-independent growth of esophageal and lung cell lines. SOX2-driven tumors expressed both squamous differentiation and pluripotency markers which introduced SOX2 as a lineage-survival oncogene in SCC of both lung and esophagus (Bass et al., 2009). Nevertheless, the exact regulation of SOX2 in pathway-dependent pluripotency and tumorigenesis has not been fully addressed yet.

LncRNAs have been suggested to regulate the expression of neighboring overlapped or antisense genes via different mechanisms (Mercer et al., 2009; Hung and Chang, 2010). The location of *SOX2* gene within the intronic region of *SOX2OT* gene proposed a possibility for SOX2 expression regulation by SOX2OT. This hypothesis is more approved by several experimental approaches obtained from gene expression alteration during stem cell differentiation or carcinogenesis, and also by manipulation of SOX2OT expression *in vitro* (Amaral et al., 2009; Askarian-Amiri et al., 2014; Hou et al., 2014; Shahryari et al., 2014). Similar dynamic regulation of sox2ot transcripts and sox2 proposed a conserved role for sox2ot in vertebrate embryogenesis and neuronal system development (Amaral et al., 2009).

Using the RNA interference strategy, our group performed a functional assay on SOX2OT, where the data supported our hypothesis on the existence of a positive regulation of SOX2 and OCT4 by SOX2OT (Shahryari et al., 2014). In line with the data, Askarian-Amiri et al. (2014) demonstrated that ectopic expression of SOX2OT caused increased SOX2 expression level. They also demonstrated that the enriched suspension culture of breast cancer cells, which favors stem cell growth, exhibited upregulation of both SOX2 and SOX2OT expression, in comparison to the original adherent cells (Askarian-Amiri et al., 2014).

Furthermore, SOX2OT exerts regulatory function in cell cycle progression; hence its association with carcinogenesis of human tumors of breast (Askarian-Amiri et al., 2014), esophagus (Shahryari et al., 2014), and lung (Hussenet et al., 2010; Hou et al., 2014) cancers is not surprising. SOX2OT controls lung cancer cell proliferation, and represents a novel prognostic indicator for this cancer (Hou et al., 2014). The knocking down of SOX2OT caused induction of G2/M arrest, prohibition of S phase entry and inhibited cell proliferation which correlated with reduced protein levels of Cyclin B1 and Cdc2 in human lung cancer cell lines. SOX2OT moderated lung cancer cell cycle progression through regulating EZH2 expression level; albeit any evidence of physical interaction between them has not been observed (Hou et al., 2014). EZH2 (a histone-lysine *N*-methyltransferase enzyme) is a major component of the polycomb repressive complex 2 (PRC2) which is involved in maintaining the transcriptional repressive state of its target genes (Cardoso et al., 2000; Cao et al., 2002).

High expression levels of SOX2OT and SOX2 are associated with estrogen receptor status and tamoxifen sensitivity of breast cancer cells (Askarian-Amiri et al., 2014). SOX2OT and SOX2 co-upregulation has been reported in lung tumor tissues, particularly in squamous cell lung carcinoma (Hussenet et al., 2010; Hou et al., 2014), which is related to 3q26.33 genomic amplification (Hou et al., 2014). A statistically significant correlation coefficient between SOX2 and SOX2OT in cancer tissues (Askarian-Amiri et al., 2014; Hou et al., 2014; Shahryari et al., 2014), suggested the possibility of SOX2OT role in the regulation of SOX2 expression.

Altogether, current evidences indicate a functional association between SOX2OT and SOX2 in tumorigenesis, cellular differentiation, and pluripotency (Table 1). Yet, more remains to be investigated on the mechanisms underlying this regulation.

**TABLE 1:** **Recent studies which highlighted emerging roles of SOX2OT in pluripotency and carcinogenesis**.

Reference	SOX2OT association with pluripotency or carcinogenesis	SOX2OT status
Amaral et al. (2009)	Vertebrate embryos	Upregulated
	Embryonic body differentiation	Downregulated
Hussenet et al. (2010)	Squamous cell carcinoma of lung with 3q.26 amplification	Upregulated
Askarian-Amiri et al. (2014)	Estrogen positive to estrogen negative breast cancer tissue	Upregulated
	Tamoxifen resistant breast cancer cell lines	Downregulated
	Suspension culture enriched breast cancer cell	Upregulated
Shahryari et al. (2014)	Esophagus squamous cell tumor to normal tissue	Upregulated
	NT2 neuronal like differentiation	Downregulated
Hou et al. (2014)	Lung cancer of squamous and adenocarcinoma to normal tissue	Upregulated

## Concluding Remarks

According to recent achievements, a large number of lncRNAs primarily exert their biological functions through induction of epigenetic events including DNA methylation or histon modifications in their target genes. This is mediated by the well-known chromatin modifying complexes of PRC1 and PRC2, as well as other related complexes in a *cis-* or *trans*- acting manner (Prensner and Chinnaiyan, 2011; Wang and Chang, 2011; Brockdorff, 2013). Multiple lncRNAs including HOTAIR, ANCR, and ANRIL are able to recruit PRC1 or PRC2 complexes to genomic regulatory regions of their target genes to reshape/regulate the chromatin state/their expression (Gupta et al., 2010; Aguilo et al., 2011; Kretz et al., 2012).

LncRNA ANRIL is involved in various mechanisms of epigenetic regulation including triggering a repression of INK4 locus by SUZ12 in PRC2 (Kotake et al., 2011), an induction of chromatin silencing of the CDKN2A/B genes through interaction with CBX7 in PRC1 (Yap et al., 2010), and an alteration of DNA methylation of the locus in differentiated cells (Yu et al., 2008). Genomic association of *SOX2* and *SOX2OT* remarkably resembles that of *ANRIL* and *CDKN2B*. Similarly, the lncRNA *ANRIL* holds the protein-coding gene *CDKN2B* in its intronic region, albeit in the antisense/opposite strand.

A brain specific lncRNA known as RMST which is involved in modulating neurogenesis physically interacts with SOX2. By acting as a transcriptional coregulator, RMST helps SOX2 to bind to regulatory regions of that of target genes which have a role in the regulation of neural stem cell fate (Ng et al., 2013). Although recent studies on SOX2OT and SOX2 have not claimed the existence of any physical interaction between them, the functional assays obtained from both knockdown and overexpression events have demonstrated that SOX2OT has a positive effect on SOX2 expression (Askarian-Amiri et al., 2014; Shahryari et al., 2014). As it was mentioned above, SOX2OT regulated the expression of EZH2 (in PRC2); however, the exact mechanism of regulation of SOX2 expression by SOX2OT mediated either by regulating PRC2 or other molecular mechanism remained largely questionable.

Several isoforms of Sox2ot which originated from alternative TSSs are associated with chromatin modifications characteristic of well-known promoters in HCEs. These isoforms have tissue or cell type specific signature, and are differentially regulated (Kimura et al., 2006; Denoeud et al., 2007; Amaral et al., 2009). This event is more prominent in SOX2DOT isoform which has a specific tissue expression pattern restricted to the adult mouse brain. SOX2DOT also demonstrates different expression patterns during differentiation of ESCs and neurospheres. The existence of alternative splicing and alternative TSSs suggests that the different transcripts of Sox2ot might have differential regulation and function (Amaral et al., 2009).

Moreover, according to the sequences registered for SOX2OT in EST database of NCBI, it is deduced that SOX2OT could have more than three splicing variants with a unique tissue or cell type specific expression signature. Moreover, the isoform of SOX2DOT indicates a more complex splicing pattern for SOX2OT. Altogether, the overlapped expression of SOX2OT with SOX2, and the conserved association between them in different developmental systems of vertebrates, and also in human cancer and stem cells all support the existence of a complex functional regulatory relationship. The latter could be a consequence of having similar regulatory elements that regulate the expression of both Sox2ot and Sox2 (Amaral et al., 2009; Askarian-Amiri et al., 2014; Shahryari et al., 2014).

Several conserved genomic regions upstream of *SOX2OT* and *SOX2DOT* serve as the binding sites for key transcription factors responsible for controlling the pluripotency as well as tumorigenesis processes. This observation along with the observed correlations between the expression of SOX2OT variants with that of key genes promoting those events, all suggested a key role for SOX2OT in pluripotency and tumorigenesis.

In this review we have provided insights into structural characteristics, epigenetic modifications, and splicing patterns of *SOX2OT* gene. Furthermore, the expression patterns of its variants and their emerging roles in stem cell biology and tumorigenesis is discussed. It is clear that SOX2OT has a positive regulatory effect on SOX2 expression; however, the exact molecular mechanism remains to be elucidated. Specifying SOX2OT-dependent molecular pathways in organ tissue culture or engineered animal models may identify more common pathways between development, pluripotency and tumorigenesis.

In conclusion, current evidences support the idea that the lncRNA SOX2OT is a key regulatory molecule in mediating pluripotency and tumorigenesis events, probably through regulation of SOX2 expression. The positive effect of SOX2OT upon SOX2 expression also supports a role for it in promoting generation of iPSCs. SOX2OT has a potential to be employed as a novel prognostic indicator/therapeutic target of several human cancers including breast, lung and esophagus cancers.

### Conflict of Interest Statement

The authors declare that the research was conducted in the absence of any commercial or financial relationships that could be construed as a potential conflict of interest.
